# Systematic Monitoring of Male Circumcision Scale-Up in Nyanza, Kenya: Exploratory Factor Analysis of Service Quality Instrument and Performance Ranking

**DOI:** 10.1371/journal.pone.0101235

**Published:** 2014-07-01

**Authors:** Dickens S. Omondi Aduda, Collins Ouma, Rosebella Onyango, Mathews Onyango, Jane Bertrand

**Affiliations:** 1 School of Public Health and Community Development, Maseno University, Maseno, Kenya; 2 Department of Biomedical Sciences and Technology, School of Public Health and Community Development, Maseno University, Maseno, Kenya; 3 Department of Public Health, School of Public Health and Community Development, Maseno University, Maseno, Kenya; 4 FHI360, Kisumu Office, Kisumu, Kenya; 5 Department of Global Health Systems and Development, Tulane University. New Orleans, Louisiana, United States of America; Johns Hopkins Bloomberg School of Public Health, United States of America

## Abstract

**Background:**

Considerable conceptual and operational complexities related to service quality measurements and variability in delivery contexts of scaled-up medical male circumcision, pose real challenges to monitoring implementation of quality and safety. Clarifying latent factors of the quality instruments can enhance contextual applicability and the likelihood that observed service outcomes are appropriately assessed.

**Objective:**

To explore factors underlying SYMMACS service quality assessment tool (adopted from the WHO VMMC quality toolkit) and; determine service quality performance using composite quality index derived from the latent factors.

**Study design:**

Using a comparative process evaluation of Voluntary Medical Male Circumcision Scale-Up in Kenya site level data was collected among health facilities providing VMMC over two years. Systematic Monitoring of the Medical Male Circumcision Scale-Up quality instrument was used to assess availability of guidelines, supplies and equipment, infection control, and continuity of care services. Exploratory factor analysis was performed to clarify quality structure.

**Results:**

Fifty four items and 246 responses were analyzed. Based on *Eigenvalue* >1.00 cut-off, factors 1, 2 & 3 were retained each respectively having eigenvalues of 5.78; 4.29; 2.99. These cumulatively accounted for 29.1% of the total variance (12.9%; 9.5%; 6.7%) with final communality estimates being 13.06. Using a cut-off factor loading value of ≥0.4, fifteen items loading on factor 1, five on factor 2 and one on factor 3 were retained. Factor 1closely relates to preparedness to deliver safe male circumcisions while factor two depicts skilled task performance and compliance with protocols. Of the 28 facilities, 32% attained between 90^th^ and 95^th^ percentile (excellent); 45% between 50^th^ and 75^th^ percentiles (average) and 14.3% below 25^th^ percentile (poor).

**Conclusion:**

the service quality assessment instrument may be simplified to have nearly 20 items that relate more closely to service outcomes. Ranking of facilities and circumcision procedure using a composite index based on these items indicates that majority performed above average.

## Background

Hitherto, a precise definition of quality remains elusive [1] although there is consensus about its multidimensionality as a service production variable [2]. The Institute of Medicine (IOM) defines quality as “the degree to which health services for individuals and populations increase the likelihood of desired health outcomes and are consistent with current professional knowledge” [3]. Inherent in this definition are dimensions related to health experiences and anticipated outcomes for individuals and people-groups. Guidelines for program implementation ought to address how these dimensions can be correctly assessed across diverse care settings. Accordingly valid data tools are needed to capture objective service quality information during routine program practice. This would enhance operational decision-making [4], [5], [6]; determine scope for resource allocation and improvement tasks [5], [7]; enhance accountability for service delivery tasks planned or accomplished; guideline revisions [8], [9]; and facility accreditation [10]. Furthermore, the structure of data tools should be designed to improve their effectiveness [5], [11], [12], [13].

Voluntary medical male circumcision (VMMC) service delivery occurs across multiple service levels in diverse contexts which pose considerable potential for variability in service quality [14], [15], [16], [17], [18], [19], [20], [21]. Specific issues relate to lack of adherence to guidelines [22], [23]; low level support supervision; constrained documentation [24], [25], reporting and uptake of feedback [13], [14], [17], [18]. These have implications on service uptake, safety and improvement decisions [5], [26]. Furthermore, hurdles in service quality assessment occur in relation to conceptualizing comprehensive measures [5], [13]; conducting appropriate evaluations; aggregating and reporting assessment information; attributing variability to specific service quality measures and [27]; applying the results to improve program goals [10].

Considering these challenges and as part of efforts to ensure sustained quality of services, the national VMMC program adapted for use in 2009 [16], [28], [29], [30] the World Health Organization (WHO) toolkit [6] for monitoring a range of quality standards at facility level. The instrument is a comprehensive checklist comprising of 10 standards and 36 criteria. Additionally, the Kenya Quality Model for Health (KQMH) which was launched in 2012 provides a broad framework for sectoral integration of service quality improvement and management.

While the WHO quality toolkit is a useful guide for internal and external assessment of VMMC service activities across multiple levels, there is need to make it more user-friendly and assess its use across different service dimensions and locale since quality presentations may be influenced in part by constructs that relate closely to cultural context [13]. Hitherto, anecdotal field reports indicate that the toolkit is too laborious and the information collected is often complex to interpret, making its use problematic.

This paper applies exploratory factor analysis (EFA) to simplify the quality instrument, elicit variable interrelationships, identify latent dimensions and clarify content structure [31]. The principal factors explaining substantial variability is used to construct a quality index. This is a composite of the observed items that can be used for routine service quality assessment.

The aims include: *(i)* exploring the underlying dimensions and interrelationships of the items comprising the SYMMACS service quality assessment tool (adopted from the WHO quality toolkit [6]);*(ii)* identify key constructs which demonstrate optimal performance;(*iii)* to derive a quality index based on the observed quality factor scores and use it to categorize performance of the service facilities. The study outcomes can be adopted by team managers for routine service quality assessment.

## Materials and Methods

### Study design

This was comparative process evaluation of the voluntary medical male circumcision scale-up in Kenya over two years. The SYMMACS, Systematic Monitoring of the Medical Male Circumcision Scale-Up, quality instrument was used to assess respective facilities providing VMMC for availability of guidelines, supplies and equipment, infection control, and continuity of care services as well as direct observation of VMMC surgeries.

#### Context of primary study and data collection

The SYMMACS study was conducted in Kenya (Nyanza region), Tanzania, Zimbabwe and South Africa. One of the objectives included evaluating evolution of safety and efficiency during VMMC scale-up, from the management perspective. The SYMMACS service quality assessment tool used was adopted from the WHO quality toolkit, which also serves as the national reference for VMMC service quality evaluation [21]. While comprehensive voluntary medical male circumcision is a standard-based HIV prevention service, no systematic evaluation of service quality has been conducted since its roll-out in 2008. In Kenya, the study provided the first opportunity for the national program to systematically evaluate performance.

#### Sampling and data collection methodology

Bertrand and colleagues [24] have described in details the SYMMACS sampling procedure. Thirty fixed, outreach or mobile VMMC sites (15/12/3) out of the 235 operational by December 2010 were randomly selected for 2011 data collection. In 2012 four of the outreach sites were replaced because of programmatic changes and one outreach site was dropped for lack of clients, resulting in 29 sites in 2012. The four facilities were respectively replaced with those of similar categories by randomly selecting from among the functional sites in the original sampling frame. Field staff sampled all clinical VMMC service providers per site over two days of data collection (a total of 86 in 2011 and 82 in 2012). Ten VMMC procedures were observed per site where feasible, starting with the first operation on Day 1 and continuing with each subsequent one available for observation. In total, 151 and 218 circumcisions were observed in 2011 and 2012 respectively.

#### Measure instruments and measures

Site level data was collected using the SYMMACS quality assessment tools, modified from the WHO quality assessment toolkit. The aspects considered were availability of guidelines, supplies and equipment, infection control, and continuity of care services. Specifically, 29 variables targeted the facility service setting and another 29 items the circumcision procedure [25]. A total of 167 clinical providers were interviewed and 369 circumcisions observed. With at least 50 variables and factor loadings of 0.40 required, the sample size obtained was considered sufficient to produce stable outcomes.

### Statistical Analysis

Binary interval data from the two instruments were merged and analyzed using SAS (SAS Institute Inc. USA). Principal component analysis (PCA) was used to identify fewer variables which account for the most variability observed in the dataset, based on the variance within and correlation across the variables [32], [33], [34], [35]. In this process, after the first component is defined, consecutive components are extracted from each subsequent residual variance until virtually all variance of measured items are accounted for.

**Figure 1:**
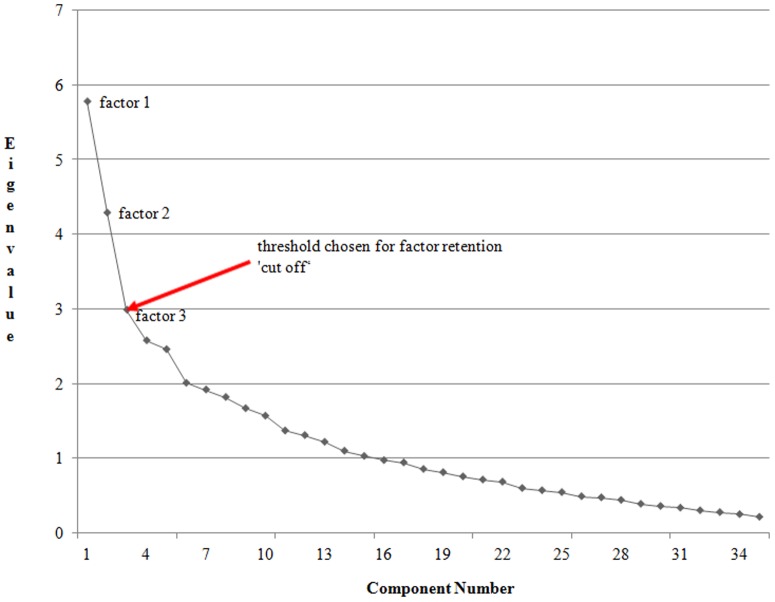
Scree plot showing distribution of factors by their eigenvalues. A Scree plot of eigenvalues of the unrotated factors displaying an ‘elbow’ of the plot (shown by the red arrow). This point of the curve represents the threshold chosen for retention of the initial factors extracted from the observed variables and which maximize the variance accounted for. Three factors, each respectively with eigenvalues of 5.78; 4.29; 2.99 were retained. These factors cumulatively accounted for 29.1% of the total variance (12.9%; 9.5%; 6.7%) with final communality estimates being 13.06. The shallow “scree” distal to the arrow demonstrates small extent of variance accounted for by the subsequent minor factors, which were deleted.

A Screeplot of the eigenvalues of unrotated factors displays a steep “cliff” of the curve representing the initial factors extracted (‘latent variables’ or constructs) from the observed variables and which maximize the variance accounted for (Fig.1), while the shallow “scree” demonstrates small extent of variance accounted for by the subsequent minor factors [36]. Conventionally, the cut-off point is where the slope forms an ‘elbow’, being ‘the point at which the slope approaches zero’. Factors with values above this point are retained while those below it are deleted given the variance accounted is almost zero [35], [37]. Exploratory factor analysis was used to examine the latent structure of extracted components and identify associations among multiple variables comprising each one [34], [35].

#### Rotating factors

to simplify the structure of the variables, Varimax rotation was used since it maximizes the variability of loadings between factors. Simple, meaningful structure is achieved when items cluster exclusively or highly on as few of the retained factors as possible, but primarily one [34].

#### Factor loading

The maximum number of iterations was set at 25 to identify variables in each dimension. Factor loadings with absolute values ≥ 0.4were considered to contribute sufficiently to the overall variability accounted for by the factor [38], [39]. Cross loading items with values >0.3 were removed to improve consistency.

Quality weights for constructing the index was obtained from the first component as it accounted for the most variability in the items observed [33]. The constructed quality index was used to rank the facilities as being excellent; good; average and poor based respectively on the cut off scores corresponding to the 90^th^, 75^th^, 50^th^ and 25^th^ percentiles (0.867; 0.491;−0.219and −0.667).

### Ethical Considerations

"Ethics approval for the SYMMACS study was obtained from the Tulane University Institutional Review Board (IRB) and local IRB, the Kenya Medical Research Institute. Academic approval was obtained from Maseno University. All study participants provided written informed consent."

## Results

### Principal component analysis

A total of 54 item measures and 246 responses with normal distribution were analyzed. Based on a stepwise approach highly correlated items cluster around respective common factors (latent variable), such that the first few components/factors successively account for most of the variation in the original observed set of variables and are retained to form new dimensions for the measures. The initial estimate of common variance among all the 58 factors was 45, accounting for 77.6% of the total variance. Fifteen components with eigenvalues ≥1.00 accounted for 73.6% of the total variance. Based on *eigenvalue>1.00* [35] three factors, each respectively with eigenvalues of 5.78; 4.29; 2.99 were retained (Fig. 1). These factors cumulatively accounted for 29.1% of the total variance (12.9%; 9.5%; 6.7%) with final communality estimates being 13.06.

Respective factor items with values ≥0.4 are displayed list-wise in table 1. Based on this cut off, fifteen (15) items loading on factor 1, five (5) on factor 2 and one (1) on factor 3 were retained.

**Table 1:** Rotated factor loadings of factor 1 and 2 relating to VMMC service quality dimensions.

Variables	Factor 1 [preparedness]	Factor 2 [performance-safety]	Factor 3
Basic life support equipment (CPR) is on hand in case of an emergency -Antihistamine.	**0.67**	0.16	0.30
Staff reviews vital signs.	**0.64**	0.32	−0.04
Staff observes post-op clients for an allergic reaction or any other abnormality before allowing them leave the operating table or recovery room.	**0.62**	0.33	−0.37
Basic life support equipment (CPR) is on hand in case of an emergency:- IV Lines.	**0.60**	0.13	0.39
Appropriate antibiotics in stock to treat infection related AEs.	**0.58**	−0.15	**0.44**
Basic life support equipment (CPR) is on hand in case of an emergency:-Bag &mask.	**0.57**	**0.51**	0.30
WHO guidelines for performing MC or National guideline of Standard Operating Procedure (SOP) for VMMC available on site.	**0.53**	−0.35	0.34
Clinical personnel conduct a basic preoperative examination.	**0.52**	−0.13	0.09
Patients receive post -operative counseling instructions and reinforcement of previous VMMC/HIV messaging.	**0.51**	0.36	−0.23
National protocols for syndromic management of STIs available on site.	**0.51**	−0.20	0.38
Facility offers HIV testing and counseling (HTC).	**0.49**	−0.73	−0.22
Staff provides private individual counseling and question time on VMMC and offers HTC.	**0.49**	−0.73	−0.22
Male condoms available for distribution to clients.	**0.48**	−0.15	−0.27
Staff provide patients with clear instructions, (verbal and written) on how to wash and care for the wound and how to deal with pain and minor bleeding	**0.46**	0.25	−0.21
Uses correct technique in tying surgical knots.	**0.41**	0.21	−0.35
Basic life support equipment (CPR) is on hand in case of an emergency: - Oxygen supply.	−0.23	**0.71**	0.31
Use of protective eyewear by all providers.	0.29	**0.51**	0.30
Staff gives specific reminders of the 6 week post of the 6 week post-operative abstinence period.	−0.11	**0.46**	−0.25
Staff insist/encourage clients to return for at least one follow up visit or in case of a complication.	0.29	**0.51**	−0.47

*CPR =  cardiopulmonary resuscitation; VMMC =  voluntary medical male circumcision; STI =  sexually transmitted infections; HTC =  HIV testing and counseling. Bolded values represent loadings between 0.4 and 0.7.*

Factor one items relate to different aspects of VMMC service delivery quality indicators, focusing broadly on the preparedness to provide VMMC in terms of physical infrastructure, guidelines and the interactive elements of circumcision service. Hence it is labeled ‘preparedness to deliver safe male circumcisions’. Items converging on factor 1 can be categorized further into: *safety reliability* (availability of basic life support equipment, eligibility assessment, observation of vital signs and other events post-operatively to identify potential harms and, availability of antibiotics for treatment of adverse events); *appropriateness* (using guidelines in performing necessary pre-, intra- and post-operative tasks);*communication interaction*(pre- and post- operative information-giving on HIV and circumcision); *access to minimum service package* (Syndromic management of STIs, individualized confidential HTC and condom distribution) and *staff competence* (correct surgical knots tying technique).

Factor 2 is labeled ‘performance-safety’ being related to skill-compliance issues and safety of surgical procedure. It comprises of variables related to *continuity of care* (discharge care and interactive follow-up instructions); staff *safety* (eye wear to prevent splash to the eyes); *acute care* (Oxygen as a basic life support). The only item loading on factor 3 retained based on the threshold for cut off was *‘Appropriate antibiotics in stock to treat infection related AEs’.* However, it was also cross-loading on factor 1. Since factor three had only one item, also cross-loading on factor 1, it was considered weak hence was not considered further.

### Quality of care by facility based ranking by composite quality index derived from the principal factors

The weighted factor coefficients from the first component were used to rank facilities and the cases performed (Table 2). Out of the 28 facilities, 32% (9/28) had scores between 90^th^and 95^th^ percentile; and 45% between 50^th^and 75^th^percentiles. In four of the facilities, scores for all cases observed were poor, being in the lower 25^th^ percentile. Quality scores for more than half of the cases were rated as good or excellent, while almost a quarter of them were poor [table 3].

**Table 2:** Overall facility ranking by weighted quality scores.

Facility Identification #	Average facility index score	Percentile
111	1.325	≥90^th^ percentile
101	1.288	
112	1.246	
133	1.244	
103	1.174	
107	1.168	
125	1.098	
134	1.059	
131	0.980	
102	0.429	75^th^ percentile
109	0.417	
123	0.275	
114	0.223	
126	0.201	
104	0.155	
106	−0.053	
110	−0.065	
136	−0.107	
121	−0.432	50^th^ percentile
130	−0.563	
118	−0.564	
132	−0.645	
108	−0.774	25^th^ percentile or lower
119	−0.980	
137	−1.103	
129	−1.201	
105	−1.500	
124	−2.230	

**Table 3:** Service quality ranking by cases in 2011 and 2012 and by facility in 2012.

	Cases performed				Overall facility ranking
	2011		2012		2012
	Freq.	%	Freq.	%	%
Excellent	22	24.4	71	32.6	32
Good	23	25.6	57	26.2	32
Average	22	24.4	41	18.8	14
Poor	23	25.6	49	22.5	21
Total	90	100	218	100	100

## Discussions

The factor analysis of the SYMMACS quality instrument reveals two discreet factors. The value of extracted factors in measuring service quality is however contingent on the observed data and the relationships between variables under consideration as well as the validity and reliability of the variables retained [40]. It is to be noted that rotation of factors to simplify structure may cause loss of variance on individual dominant sources [40].

The factor loadings show the hierarchical item importance within the factors, in terms of both component availability and task performance [34]. The observed variability in quality of VMMC service delivery is best explained by the dimensions *‘preparedness to deliver safe male circumcisions’*, being complemented by *‘performance-safety’*. Implicit in these dimensions are the technical and functional requirements necessary for accomplishing VMMC service delivery tasks correctly. The current study was undertaken from the management perspective to objectively evaluate provider performance, whereas existing studies on health service quality largely focus on the consumers' viewpoint, which is less likely to reflect accurately aspects like provider competence [41], [42], [43], [44].

The SYMMACS quality instruments exhibit similar underlying concepts as those described in existing health service quality studies [5], [44], [45], [46], [47], [48]. The factors configure well with the WHO toolkit criteria [6], [49] for assessing VMMC service quality and the domains (Effective, Appropriate, Safe, Efficient, Responsive, Accessible, Continuous, Capable, Sustainable) in other health systems quality performance frameworks [7], [50]. The principal component represents diversity of service quality sources and reflects the multidimensionality of VMMC services similar to other public health interventions.

The factor structure elicited demonstrates safety aspects and provider-client interactions as key quality considerations. This would potentially assist program managers to understand the scope encompassed by VMMC quality assessment tool, recognize its importance and progressively build into the delivery system capacities for proper service performance. The observations also indicate that staff performed dismally mainly in the tasks related to spontaneous patient-staff communication interactions, particularly engagement in the post operative period in contrast to availability of equipment and supplies which by default are provided by the program [25]. These performance tasks are inherently related to individual competency and responsiveness which if emphasized would greatly improve patient safety.

As part of quality improvement plans, operational guidelines ought to subsequently clarify systemic approaches to health practice safety. Likewise, emphasizing compliance with operational guidelines will ensure desired service quality outcomes are obtained [6], [43]. Refresher staff training and support supervision are helpful in enhancing progressive learning of skills for target tasks and responsibilities besides monitoring how well these are performed [43], [47], [51]. These capacities includes communication abilities and interpersonal skills to improve information-giving [52].

Historically, service quality has been assessed via similar basic dimensions [43], [44]. The Donabedian framework, for example, proposes ‘structure – process – outcome’ approach [47]. In this approach, the structure dimension relates to the care setting (including facility characteristics, equipment, training and special skills). The process dimension comprises aspects related to the provider-patient interaction and is considered a function of the technical and interpersonal skills. The outcome dimension reflects the immediate/intermediate and long-term changes occurring to the patient's status based on services provided [3], [8], [12], [53]. Another model developed by Parasuraman *et al.* (1985) has 10 dimensions: tangibles, reliability, responsiveness, competence, courtesy, credibility, assurance, access, communication, and customer understanding. Brown and Swartz (1989) applied this model to assess quality of medical-surgical service delivery and they determined that this list is reasonably applicable to health service settings. Bruce-Jain's framework however, consists of six dimensions: needs assessment, choice of contraceptive methods, information given to users, interpersonal relations, constellation of services, and continuity mechanisms. This has been used to assess contraceptive services to adolescents in Uganda [49]. Given the dimensions apparent in the SYMMACS service quality instruments are reasonable, a simplified version based on the principal factors can be adopted for routine quality assessment and monitoring.

## Conclusion

Using exploratory factor analysis, it was possible to empirically discern the multidimensionality of VMMC service delivery by eliciting three principal factors of service quality. Whereas the quality assessment tool contains globally useful items, only twenty of these were more closely related to service quality performance outcomes in the local context. Future research should focus on defining other conceptually different item combinations for the toolkit.

The observed factor structure can be a realistic guide to quality performance improvement efforts despite inherent potential limitations related to its structure. In this study, performance of majority of facilities was rated as above average based on the derived composite quality index scores, indicating respective level of improvement efforts needed.

## Study Limitations

A key limitation of the study is lack of the client perspective. However, it is unknown how its inclusion would alter the characteristics of the derived factor constructs, given that theoretically, this aspect as an outcome is difficult to link with the structure and process that produce it unless comprehensive reliable information is available.
